# Change in the Body Mass Index Distribution for Women: Analysis of Surveys from 37 Low- and Middle-Income Countries

**DOI:** 10.1371/journal.pmed.1001367

**Published:** 2013-01-15

**Authors:** Fahad Razak, Daniel J. Corsi, SV Subramanian

**Affiliations:** 1 Faculty of Medicine, University of Toronto, Toronto, Canada; 2 Harvard Center for Population & Development Studies, Harvard University, Cambridge, Massachusetts, United States of America; 3 Department of Society, Human Development and Health, School of Public Health, Harvard University, Boston, Massachusetts, United States of America; University of Bristol, United Kingdom

## Abstract

**Background:**

There are well-documented global increases in mean body mass index (BMI) and prevalence of overweight (BMI≥25.0 kg/m^2^) and obese (BMI≥30.0 kg/m^2^). Previous analyses, however, have failed to report whether this weight gain is shared equally across the population. We examined the change in BMI across all segments of the BMI distribution in a wide range of countries, and assessed whether the BMI distribution is changing between cross-sectional surveys conducted at different time points.

**Methods and Findings:**

We used nationally representative surveys of women between 1991–2008, in 37 low- and middle-income countries from the Demographic Health Surveys ([DHS] *n* = 732,784). There were a total of 96 country-survey cycles, and the number of survey cycles per country varied between two (21/37) and five (1/37). Using multilevel regression models, between countries and within countries over survey cycles, the change in mean BMI was used to predict the standard deviation of BMI, the prevalence of underweight, overweight, and obese. Changes in median BMI were used to predict the 5th and 95th percentile of the BMI distribution. Quantile-quantile plots were used to examine the change in the BMI distribution between surveys conducted at different times within countries. At the population level, increasing mean BMI is related to increasing standard deviation of BMI, with the BMI at the 95th percentile rising at approximately 2.5 times the rate of the 5th percentile. Similarly, there is an approximately 60% excess increase in prevalence of overweight and 40% excess in obese, relative to the decline in prevalence of underweight. Quantile-quantile plots demonstrate a consistent pattern of unequal weight gain across percentiles of the BMI distribution as mean BMI increases, with increased weight gain at high percentiles of the BMI distribution and little change at low percentiles. Major limitations of these results are that repeated population surveys cannot examine weight gain within an individual over time, most of the countries only had data from two surveys and the study sample only contains women in low- and middle-income countries, potentially limiting generalizability of findings.

**Conclusions:**

Mean changes in BMI, or in single parameters such as percent overweight, do not capture the divergence in the degree of weight gain occurring between BMI at low and high percentiles. Population weight gain is occurring disproportionately among groups with already high baseline BMI levels. Studies that characterize population change should examine patterns of change across the entire distribution and not just average trends or single parameters.

*Please see later in the article for the Editors' Summary*

## Abstract 2

Using cross-sectional surveys, Fahad Razak and colleagues investigate how the BMI (body mass index) distribution is changing for women in low- and middle-income countries.

## Editors' Summary

**Background:**

The number of obese people (individuals who have an excessive amount of body fat) is rapidly increasing in many countries. Globally, there were about 200 million obese adults in 1995; by 2010, 475 million adults were obese and another billion were classified as overweight. Obesity is defined as having a body mass index (BMI, an indicator of body fat calculated by dividing a person's weight in kilograms by their height in meters squared) of more than 30.0 kg/m^2^. Compared to people with a healthy weight (a BMI between 18.5 and 24.9 kg/m^2^), obese individuals and overweight individuals (who have a BMI between 25.0 and 29.9 kg/m^2^) have an increased risk of developing diabetes, heart disease and stroke, and tend to die younger. At the same time in many developing countries substantial numbers of people are underweight (BMI <18.5 kg/m^2^) or have chronic energy deficiency (BMI <16.0 kg/m^2^) and are at risk of increased risk of dying due to infectious disease or respiratory problems.

**Why Was This Study Done?:**

The global obesity epidemic is usually described in terms of increases in the average BMI or in the prevalence of obesity (the proportion of the population whose BMI is above 30.0 kg/m^2^). Such descriptions assume that the BMIs of fat and thin people are increasing at the same rate and that the shape of the population's BMI distribution curve remains constant. However, as average BMI and the prevalence of obesity can increase it is unclear how the prevalence of underweight changes. This is potentially important for the health of the population because underweight individuals, like obese individuals, tend to die younger than healthy weight individuals, particularly in low-income countries. In this study, the researchers use repeated cross-sectional survey data collected from low- and middle-income countries in the Demographic and Health Surveys (DHS) to examine changes in BMI in women across the BMI distribution between 1991 and 2008. Repeated cross-sectional surveys collect data from a population at multiple time points from different individuals drawn from the same population, DHS are a data collection and surveillance project that help developing countries track health and population trends.

**What Did the Researchers Do and Find?:**

The researchers used statistical models to analyze data from DHS surveys of more than 730,000 women living in 37 low- and middle-income countries (two to five surveys per country). Increasing average BMI was associated with an increase in the standard deviation of BMI (a measure of the dispersion of BMI in the population) both across and within countries over time. With increasing average BMI, the BMI at both the 5th and 95th percentile increased; 90% of the BMIs in a population lie between these percentiles so these BMI values indicate the spread of the BMI distribution. However, the BMI at the 95th percentile increased about 2.5 times faster than the BMI at the 5th percentile. Moreover, with increasing average BMI, the prevalence of overweight and obesity increased faster than the decline in the prevalence of underweight. Finally, quantile-quantile plots for each country (a graphical method that compares two distributions) revealed a consistent pattern of unequal weight gain across the BMI distribution as average BMI increased, with pronounced weight gains at the obese end of the distribution and little change at the underweight end.

**What Do These Findings Mean?:**

These findings show that increases in average BMI are associated with an increased spread of BMI across and within populations. Consequently, changes in average BMI or single measurements such as the prevalence of overweight do not capture the divergence in the degree of weight gain occurring between that part of the population that has a low BMI and that part that has a high BMI. In other words, at least for the low- and middle-income countries included in this study, population weight gain is occurring disproportionately among groups with high baseline BMI levels. The researchers suggest, therefore, that the characterization of the BMI of populations over time should examine the patterns of change across the whole BMI distribution. Moreover, rather than a single broad population strategy for weight control, optimum health outcomes, they suggest, might be achieved by a strategy that includes targeted interventions to reduce weight in high BMI segments of the population and to increase weight in low BMI segments.

**Additional Information:**

Please access these Web sites via the online version of this summary at http://dx.doi.org/10.1371/journal.pmed.1001367.

- The US Centers for Disease Control and Prevention provides information on all aspects of overweight and obesity (in English and Spanish)
- The World Health Organization provides information on obesity (in several languages); Malri's story describes the health risks faced by an obese child
- The UK National Health Service Choices website also provides detailed information about obesity and a link to a personal story about losing weight
- The International Obesity Taskforce provides information about the global obesity epidemic
- The US Department of Agriculture's ChooseMyPlate.gov website provides a personal healthy eating plan; the Weight-control Information Network is an information service provided for the general public and health professionals by the US National Institute of Diabetes and Digestive and Kidney Diseases (in English and Spanish)
- MedlinePlus has links to further information about obesity (in English and Spanish)

## Introduction

Increases in the prevalence of people who are overweight (body mass index [BMI] > 25.0) or obese (BMI > 30.0) have been documented in both high-income countries, and more recently, in low- and middle-income countries (LMICs) [1],[2]. A key aspect of the discourse describing the global patterns of weight gain has been an almost exclusive reliance on the proportion of the population above thresholds for overweight or obese, or on increases in the mean BMI. The implicit assumption in such descriptions of change is that the distribution of BMI or the dispersion in the population has remained constant, with the entire distribution moving typically to the right, allowing summaries of population change by examining a single parameter. This assumption underscored the argument laid out by Geoffrey Rose when describing patterns of population change [3]. For example, blood pressure or cholesterol exists on a continuum, and the prevalence of deviant levels of these risk factors (such as the clinical entities of hypertension or dyslipidemia) is directly related to the mean level of each risk factor. This finding also extended to BMI, and using a cross-sectional sample from the InterSalt study, the correlation between mean BMI and prevalence of overweight was 0.94 [4]. Rose postulated that for most risk factors, as the average level in the population changed the “dispersion around the average remains rather constant.” [3]. However, he noted that BMI may not follow this rule, and that a proportionally greater increase appeared to exist at tail of the BMI distribution, in those with “exceptional obesity.” This pattern of increased weight gain among higher percentiles of the BMI distribution has been noted in a few national surveys of children and adults [5],[6]. Within the United States, earlier cycles of National Health and Nutrition Examination Survey (NHANES) showed similar population patterns of divergent degrees of BMI increase, but weight gain was more equally distributed in later cycles [7]–[9].

To our knowledge, changes in the distribution of BMI within and across populations over time have not been systematically examined since Rose's original hypothesis regarding population change of risk factors. As increasing focus is placed globally on limited parameters such as the mean level of BMI or prevalence of overweight/obese [1], broader changes in the population patterning of BMI may be obscured [10]–[12]. As the mean BMI increases in a population, the level of deviance (increase at low BMI versus high BMI, or prevalence of underweight versus obese) may not change in the same way. We investigate and report the distribution and pattern of change of BMI across 37 LMICs using representative standardized cross-sectional DHS surveys of women age 20–49 y. Specifically, we answer the following questions: (1) How are changes in average BMI related to the dispersion or spread in the BMI distribution across countries, and within countries between different surveys conducted at different times? (2) With changes in mean BMI, what is the pattern of weight gain across the entire population spectrum, with specific focus on low, average, and high BMI segments of the distribution and on the categories of BMI of underweight, overweight, and obese?

## Methods

### Data Sources

Data were extracted from 96 cross-sectional surveys conducted in 37 LMICs with at least two surveys conducted since 1991 as part of the DHS project [13]. DHS are designed to collect information on population health, nutrition, and fertility and have a focus on women in the reproductive age group (15–49 y) [14]. The major strengths of DHS include standardized and representative sampling of participants across a wide range of countries with marked variation in mean BMI, objective measurement of height and weight by trained individuals using a standardized protocol, repeated surveys within countries, and high response rates [13],[15] DHS use a probability-based cluster sampling procedure, which is then adapted to specific contexts within each country [16]. First, sampling frames are developed on the basis of non-overlapping units of geography (typically census enumeration areas) that cover the entire country. These geographic areas are identified as the primary sample units (PSU); samples of which are selected with probability proportional to population size for inclusion in the survey. Next, within selected PSU a list of all households is generated through field visits and a fixed proportion of households are selected using systematic sampling [17]. Within selected households, all women or ever-married women aged 15–49 y with children under 5 y of age are invited for interviews and anthropometric (height and weight) measurement. The target number of women in this age group per PSU is typically 20–25 in urban areas and 30–40 in rural areas. In some DHS, additional women (both with and without children) who are present in selected households at the time of interview are invited to participate in the anthropometric measurement component of the survey. In our analyses, we have included all non-pregnant women with available height and weight data between the ages of 20–49 y either with or without children of any age. The household response rates of DHS typically exceed 90%.

### Study Population and Sample Size

The population included in this study is composed of non-pregnant women between the ages of 20–49 y from 37 LMIC with at least two DHS conducted where anthropometric measurements were obtained (*n* = 740,306). Survey year varied from 1991 to 2008. Although no widely applicable single definition of what constitutes an implausible value exists, criteria from previous publications on the DHS dataset were used, excluding extreme values for height (<100 cm or >200 cm) and/or weight (<25 kg or >200 kg). This resulted in exclusion of ∼1% of the population (*n* = 7,522) leaving a sample size of *n* = 732,784 used in this current analysis [18].

### Outcome, Exposure, and Covariates

The study outcome was BMI (kg/m^2^), calculated as weight (kg) divided by the square of height (m^2^). The 5th and 95th percentiles and standard deviation (SD) of the BMI distribution were used as outcomes for statistical modeling as studying these parameters provides information on how the spread of the BMI distribution changes between countries, and within countries over time. Conventional thresholds of a BMI <18.5 (underweight), 25.0–29.9 (overweight), and ≥30.0 (obese) were also modeled as outcomes. Age (in years) was included as an adjustment covariate. given the linear association of age with BMI in this dataset. Age adjustment was achieved by using age as a linear predictor of BMI across all countries, followed by the addition of the grand mean to the residuals from this model.

### Analysis

Individual country files were first created and then combined to form a pooled dataset to allow for cross-country comparisons. A second database was created containing percentile values for the BMI distribution within each country for each survey cycles. This database was used for modeling distributional changes within countries over time and between countries. SPSS version 20.0 was used for all analysis.

#### Multilevel regression modeling

Multilevel regression methods were used to account for the nested structure of the data, with repeated representative surveys conducted within countries. A standard linear regression approach that did not account for the correlation of within country data would produce biased estimates. Model fit was assessed by the pseudo-R^2^, the correlation between the predicted value from the model and the actual value form the population distribution [19]. In all analyses, BMI was age standardized as described above.

Using standard deviation as an example outcome to describe the multilevel structure, a two-level model for the standard deviation of BMI (y) in survey *i* (level 1) nested within country *j* (level 2) was estimated. Level 1 of the model is represented as:
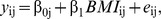
where *y*_ij_ is the standard deviation (SD) of BMI in survey *i* in country *j*, β_0j_ is the overall SD of BMI in each country, β_1_ is the slope of the relationship between mean BMI and SD of BMI, *BMI*_ij_ is the mean BMI in survey *i* in country *j*, and *e*_ij_ is the differential for each survey within countries. No random effects terms were included for β_1_ as most countries had only two or three repeated surveys and this did not provide enough samples for model convergence if β_1_ was allowed to have a random component. The level 2 model is represented as:
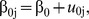
where β_0_ is the grand mean SD of BMI across all countries and *u*_0j_ is the differential of the mean SD of BMI for country *j* from the overall of SD of BMI. The level 1 and level 2 models were combined into the full multilevel model:
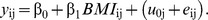



Terms inside brackets are random terms representing the between country differences in SD of BMI conditional on mean BMI in that country (*u*_0j_) and within country, between survey differences conditional on mean BMI (*e*_ij_). These terms are assumed to be independently and identically distributed and follow a normal distribution with mean 0 and variances 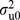 and 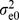 at level 2 and level 1, respectively [20].

The intraclass correlation coefficient 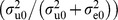 was used to estimate the amount of variance attributable to between-country differences in SD of BMI as a proportion of the total variance (sum of variance between countries and variance within countries over survey cycles) [19].

#### Analyzing specific parameters in the BMI distribution

Across all country-survey cycles, multilevel longitudinal regression models were constructed to examine the relationship between: (1) mean BMI and the outcomes of: SD of BMI, percent underweight, overweight, and obese; (2) median BMI and the outcomes of: 5th and 95th percentile of BMI. As an alternate measure of dispersion to the SD, we also modeled the coefficient of variation (SD divided by mean BMI) as Rose referenced this as a measure of dispersion that may remain constant with population change [3],[21]. Scatter-plots were also used to examine how the change in mean or median BMI from the first survey to the last survey cycle would relate to change in the level (SD, 5th and 95th percentile) or prevalence (underweight, overweight, obese) of each specific parameter.

#### Patterns of BMI distribution change between surveys

No standard approach to graphically examining distributional changes in BMI exist [8]. Quantile-quantile (QQ) plots provide a unique method of examining changes in the distribution of a variable [22]. A QQ plot is constructed by plotting percentiles of BMI at the most recent survey cycle (*y*-axis) against percentile of BMI from the baseline survey cycle (*x*-axis). If the distribution of BMI remained exactly the same, percentiles of BMI would remain the same and the QQ plot would fall on the line *y* = *x* (the line of equality). Points that are higher than the line *y* = *x* represent higher levels of BMI at the same percentile in a subsequent survey year. If everyone in the population had a uniform change in their BMI, the QQ plot would show a set deviation from the line *y* = *x*, with the slope remaining the same. Alternatively, if between two surveys high BMI segments of the population had an increase in BMI and low BMI segments of the population had little to no increase in BMI, then the QQ plot would show minimal deviation from the line of equality at low percentiles but an increasing distance from the line of equality at upper percentiles. QQ plots are especially useful in detecting deviance at the tails of the distribution [22].

To graphically examine the change in the distribution of BMI over survey cycles we used two approaches. First, in all countries, QQ plots were constructed for each country by plotting the BMI percentiles of the final survey cycle against the percentiles of the baseline survey. Second, to further explore the pattern of change, we focused on the countries that had the most repeated survey cycles within the DHS: Bangladesh, Bolivia, Egypt, Ghana, and Peru. These countries represented a wide range of baseline BMI values, with Bangladesh being in the lowest quintile of mean BMI and Egypt in the highest quintile [12]. In each of these five countries, the histogram for BMI was plotted for each survey cycle with reference lines placed for key thresholds: 16.0 (chronic energy deficiency), 18.5 (underweight), 25.0 (overweight), and 30.0 (obese) [23],[24]. QQ plots were created for each of these countries showing the change of the BMI distribution across all survey cycles. A QQ plot does not provide information on a specific percentile value (it shows the relationship between percentiles in two distributions), therefore vertical reference lines were placed to mark percentiles at the baseline survey.

### Ethical Review

The DHS was approved centrally at the ORC Macro Institutional Review Board and by individual review boards within each participating country. Oral consent was obtained from all participants. The study was evaluated by the Institutional Review Board at the Harvard School of Public Health and was considered exempt from full review as it is composed of publicly available anonymous data with no mechanism by which participants can be identified.

## Results

Table 1 presents baseline characteristics of the study population by country according to survey year. There was marked variation between countries in the distribution of BMI values. The mean BMI varied from 19.5 (95% CI 19.4–19.6) in Bangladesh (survey year 1996) to 29.5 (29.4–29.6) in Egypt (2005), 5th percentile of BMI from 15.2 in Bangladesh (2004) to 21.6 in Egypt (2005), and 95th percentile of BMI from 24.4 in Nepal (1996) to 39.7 in Egypt (2005). The highest prevalence of underweight was in Bangladesh in 1996 (38.4%) and the highest prevalence of obese was in Egypt in 2005 (42.2%).

**Table 1:** Survey year and BMI distribution parameters and prevalence of BMI <18.5 (underweight), 25.0–29.9 (overweight), and ≥30.0 (obese).

Country	Survey Year	*n*	Mean	SD	Percentiles			Prevalence [%]		
					5th	50th	95th	Underweight	Overweight	Obese
Armenia	2000	4,891	25.2	4.6	19.2	24.5	33.8	2.7	30	13.9
	2005	5,058	25.6	5.0	19.1	24.7	35.3	3	31	16
Bangladesh	1996	3,375	19.5	2.9	15.6	19.2	24.9	38.4	4.1	0.6
	1999	3,887	20.1	3.2	15.9	19.6	26.0	31.6	7.2	1.1
	2004	9,160	20.4	3.7	15.2	20.0	27.3	31.4	9.7	1.7
	2007	9,028	21.0	3.9	15.5	20.4	28.1	27	12.2	2.3
Benin	1996	2,136	21.6	3.2	17.2	21.2	27.1	12	8.8	2
	2001	4,331	22.8	4.3	17.5	22.0	31.0	9.9	15.8	6.6
	2006	12,280	22.8	4.3	17.6	22.1	30.6	9.6	15.2	5.9
Bolivia	1993	2,127	24.7	3.7	19.8	24.1	31.7	1.6	29.7	8.7
	1998	3,939	25.5	3.9	20.4	24.9	32.8	0.9	37.3	11.3
	2003	12,723	26.1	4.5	20.1	25.4	34.5	1.2	36.5	17
	2008	12,300	26.4	4.7	20.3	25.7	35.1	1.1	36.8	19.3
Burkina Faso	1992	3,189	21.6	3.4	17.3	21.2	27.2	13.4	10	1.8
	1998	3,112	21.3	3.0	17.3	21.1	26.3	14.1	6.9	1.4
	2003	8,478	21.0	3.7	16.2	20.5	27.4	23.3	7.5	2.6
Cambodia	2000	5,292	20.6	3.0	16.3	20.3	25.6	23.4	6.2	0.6
	2005	6,147	21.0	3.2	16.2	20.8	26.6	20.6	8.8	1.1
Cameroon	1998	1,426	23.6	3.9	18.4	23.0	30.4	5.3	24.1	5.4
	2004	3,467	24.1	4.4	18.3	23.5	32.3	6	24.5	10.1
Chad	1996	3,261	21.2	3.1	16.8	20.9	26.5	17.4	7.3	1.6
	2004	2,618	21.5	3.5	16.9	21.0	27.8	16.6	9.5	2.6
Colombia	1995	3,065	25.0	3.9	19.4	24.6	32.0	2.3	35.8	10.3
	2000	2,929	25.3	4.0	19.7	24.8	32.5	2.1	36.2	11.6
	2004	27,654	25.2	4.5	19.0	24.6	33.6	3.5	32.6	13.8
Cote d'Ivoire	1994	2,682	22.6	3.5	18.0	22.2	28.8	7.3	14.7	3.6
	1998	2,005	23.6	4.4	17.9	22.9	31.9	7.6	22.3	8.3
Egypt, Arab Rep.	1995	6,497	26.4	5.1	19.9	25.4	36.0	1.7	33.6	20.3
	2000	13,589	29.0	5.5	21.2	28.5	39.1	0.8	37.8	38.6
	2003	7,929	27.9	4.5	21.0	27.7	35.7	1	44.4	30
	2005	16,860	29.5	5.6	21.6	28.9	39.7	0.7	37	42.2
	2008	14,411	28.7	5.2	21.2	28.2	37.9	0.7	40.7	35.8
Ethiopia	2000	10,523	20.2	3.2	15.6	19.9	25.9	29.7	6.1	0.9
	2005	4,644	20.6	3.3	16.0	20.3	26.4	25	6.7	1.5
Ghana	1993	1,650	22.2	3.7	17.7	21.5	29.0	10.2	11.8	3.9
	1998	1,977	22.1	4.0	17.4	21.4	29.8	11.5	11.6	5
	2003	3,944	23.3	4.7	17.5	22.5	32.1	10.3	20	8.2
	2008	3,490	23.9	4.7	17.6	23.1	32.7	8.7	23.5	9.7
Guatemala	1995	4,547	24.2	3.8	19.2	23.7	31.3	2.8	28.3	7.2
	1998	2,172	25.1	4.2	19.6	24.5	33.1	2.4	34.3	11.1
Guinea	1999	2,983	22.1	3.5	17.5	21.6	28.2	11.3	12.8	2.7
	2005	2,834	21.8	3.6	16.9	21.4	28.2	15.5	12.5	2.9
Haiti	1994	1,788	21.5	3.6	16.6	20.9	28.2	17.6	11.1	2.8
	2005	3,632	22.9	4.6	16.8	22.2	31.5	14	18.9	7.2
India	1998	72,469	20.6	3.8	15.5	20.0	27.8	30.7	9.5	2.4
	2005	91,243	21.4	4.1	15.8	20.8	29.1	24.8	13.7	3.6
Jordan	1997	3,000	27.5	5.3	20.0	26.9	36.8	1.4	36.1	28.4
	2002	4,838	28.5	5.7	20.1	28.0	38.8	1.9	34.8	36.1
	2007	4,446	28.1	5.5	20.4	27.4	38.2	1.4	38	31
Kazakhstan	1995	2,900	25.0	5.2	18.3	23.9	34.9	5.9	25.8	15.6
	1999	1,880	24.2	5.0	18.3	23.2	34.2	5.6	21.6	12.2
Kenya	1998	3,009	22.4	3.7	17.8	22.0	28.8	9.4	15	3.5
	2003	5,573	23.4	4.4	17.5	22.7	31.6	10.3	22.2	8.1
	2008	6,046	23.4	4.7	17.3	22.8	32.0	11.5	21.9	8.5
Madagascar	1997	2,253	20.9	2.6	17.0	20.7	25.4	15.8	5.9	0.6
	2003	5,781	21.4	3.4	16.5	21.1	27.6	17.5	10.1	2
	2008	5,909	20.6	3.3	15.8	20.4	26.5	26.2	7.2	1.4
Malawi	1992	2,101	22.2	3.2	17.8	21.8	27.2	8.6	11.9	1.9
	2000	8,914	22.4	3.4	17.8	22.0	28.1	8.6	13.7	2.6
	2004	7,746	22.4	3.4	17.6	22.0	28.3	9.3	14.4	2.9
Mali	1995	3,787	21.5	3.1	17.2	21.2	27.2	13.2	9.1	1.7
	2001	8,415	22.3	3.8	17.3	21.7	29.4	11.6	13.4	4.3
	2006	9,774	22.7	4.3	17.3	22.0	30.7	11.7	16.5	5.9
Morocco	1992	2,795	24.1	4.4	18.6	23.3	32.3	4.5	23.8	10.3
	2003	12,713	24.7	4.4	18.5	24.2	32.8	4.9	30.7	11
Mozambique	1997	2,823	22.3	3.1	18.1	21.9	27.6	7.1	12.4	2.3
	2003	8,327	22.5	3.8	17.7	22.0	29.6	9.4	14	4.5
Namibia	1992	2,061	22.8	4.4	17.2	22.0	31.2	12	16.2	6.8
	2006	6,916	24.0	5.5	17.1	22.8	34.7	11.5	20.7	13.7
Nepal	1996	3,068	20.5	2.4	16.8	20.4	24.4	18.2	2.9	0.2
	2001	7,213	20.4	3.2	15.7	20.2	26.0	27.5	6.2	1
	2006	7,833	20.8	3.3	16.0	20.5	26.5	24.4	8.5	1.1
Nicaragua	1997	9,290	25.5	4.6	19.2	24.8	33.9	2.9	33.9	14.6
	2001	9,098	26.2	4.9	19.5	25.5	35.1	2.3	35.5	18.9
Niger	1998	2,958	21.5	3.4	17.1	20.9	28.1	15.1	9.8	2.8
	2006	3,126	22.4	4.2	17.0	21.7	30.3	13.8	16.6	5.5
Nigeria	2003	5,029	22.9	4.4	17.2	22.2	30.9	11.4	17.6	6.5
	2008	23,063	22.9	4.5	17.3	22.2	30.8	11.2	18.4	6.2
Peru	1991	4,886	24.9	3.6	20.1	24.5	31.5	1.2	35.1	8.4
	1996	10,125	25.2	3.7	20.2	24.7	31.7	1.1	37.5	9.4
	2000	20,166	25.7	4.0	20.2	25.1	33.1	1.2	38.3	13.3
	2003	20,943	25.9	4.2	20.1	25.4	33.6	1.3	38.9	15
Rwanda	2000	6,628	22.4	3.4	17.3	22.2	28.0	10.3	16	2.1
	2005	3,911	22.1	3.2	17.2	22.0	27.6	12.2	14.2	1.7
Tanzania	1996	3,502	22.3	3.4	17.7	21.8	28.5	8.6	13.1	3
	2004	7,064	22.7	4.1	17.3	22.1	30.6	11.8	16	5.9
Turkey	1993	2,294	26.5	4.8	20.1	25.8	35.1	0.9	36	21.7
	1998	2,210	26.7	4.8	20.0	26.1	35.6	1.2	37.6	21.7
Uganda	1995	2,827	22.3	3.3	18.0	21.9	28.1	7.3	12.8	2.5
	2000	4,458	22.6	3.9	17.4	22.1	29.7	10.3	16.5	4.5
	2006	1,925	22.2	3.9	17.0	21.7	29.2	13.5	15.1	4.4
Zambia	1996	3,483	22.3	3.2	17.9	21.9	27.8	8.2	13.6	2.4
	2001	5,116	21.8	3.6	16.9	21.4	28.2	14.6	11.6	2.8
	2007	4,846	22.9	4.0	17.7	22.3	30.4	9.1	17.7	5.7
Zimbabwe	1994	1,774	23.5	3.7	18.6	23.0	30.6	4.1	21.3	5.8
	2005	6,199	23.7	4.3	17.9	23.0	31.7	7.4	22.2	8.3

**Figure 1:**
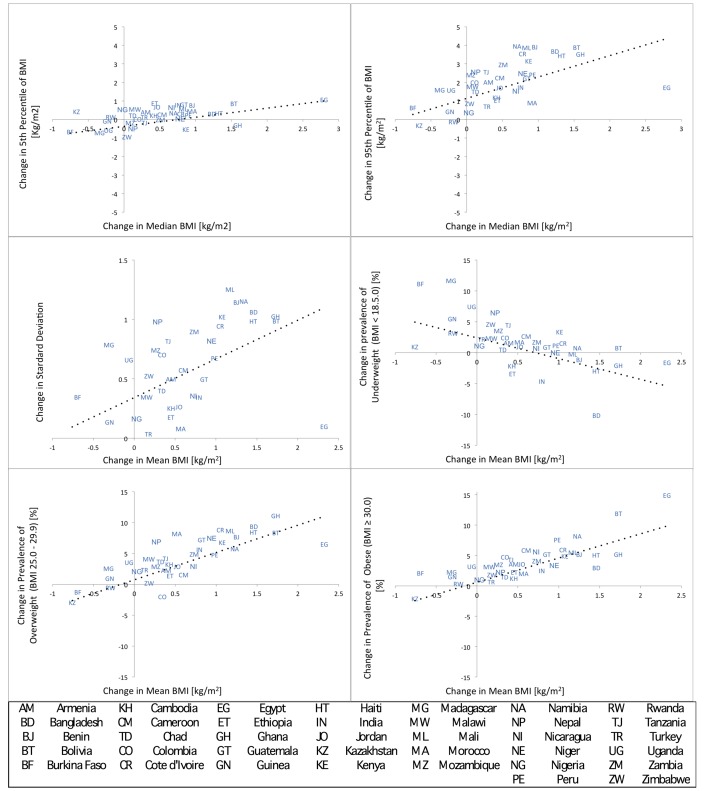
Change in specific parameters of the BMI distribution (5th and 95th percentile and SD) or categories of BMI (underweight, overweight, obese) versus change in mean or median BMI. Change is calculated from baseline survey to most recent survey and each data point is represented by a two-letter country code. Multilevel models across all country-survey cycles are contained in Table 2.

Figure 1 contains scatter-plots of the change in the BMI distribution parameters (SD and 5th and 95th percentile) or prevalence (underweight, overweight, obese) from the first survey to the last survey cycle relative to the change in mean or median BMI. β-coefficients and pseudo-R^2^ value for associated multilevel models across all survey cycles are listed in Table 2, showing moderate to strong associations with pseudo-R^2^ varying between 0.66–0.91. With rising mean BMI, there is an increase in SD that occurs both across countries and within countries over survey cycles. Reflecting the large baseline difference between countries in mean BMI and SD, 48% of the variation in SD occurs across countries and 52% occurs within countries over survey cycles. The relationship of mean BMI to coefficient of variation is also positive with a pseudo-R^2^ of 0.17.

**Table 2:** Multilevel models across all country-survey years for the relationship of median or mean BMI to each BMI distribution parameter (5th, 95th percentile, and SD) or prevalence (underweight, overweight, obese).

BMI Parameter or Category	β	95% CI	R^2^
**BMI distribution parameters**			
5th percentile	0.63^a^	(0.57–0.69)	0.90
95th percentile	1.6^a^	(1.4–1.7)	0.88
SD	0.3^b^	(0.24–0.35)	0.66
**BMI categories**			
Underweight (BMI <18.5 kg/m^2^)	−2.9^b^	(−3.4 to −2.3)	0.71
Overweight (BMI 25.0–29.9 kg/m^2^)	4.7^b^	(4.3–5.1)	0.91
Obese (BMI ≥30.0 kg/m^2^)	4^b^	(3.6–4.4)	0.88

R^2^, “pseudo-R^2^” calculated as the correlation of the prediction of the multilevel model with the actual value.

a Median BMI.

b Mean BMI.

With increasing median BMI there is an increase in both the 5th and 95th percentile of the BMI distribution. A 1.0 kg/m^2^ increase in median BMI is associated with an increase of 0.63 kg/m^2^ in the 5th percentile of BMI and a much larger increase of 1.57 kg/m^2^ in the 95th percentile of BMI (Table 2). This finding indicates that, as the median level of BMI rises, there is a continuous divergence between the tails of the BMI distribution, with the increase of BMI at the 95th percentile being approximately 2.5 times greater than the increase in the BMI at the 5th percentile. Similarly, with an increase in mean BMI the proportional increase in prevalence of overweight and obese was much greater than the decline in prevalence of underweight. For each 1.0 kg/m^2^ increase in mean BMI, the absolute prevalence of overweight increases by 4.7% and obese by 4%, relative to decline of the prevalence of underweight by 2.9% (Table 2). This represents an approximately 60% greater increase in overweight and 40% greater increase in obese relative to the decline in underweight in these societies.

**Figure 2:**
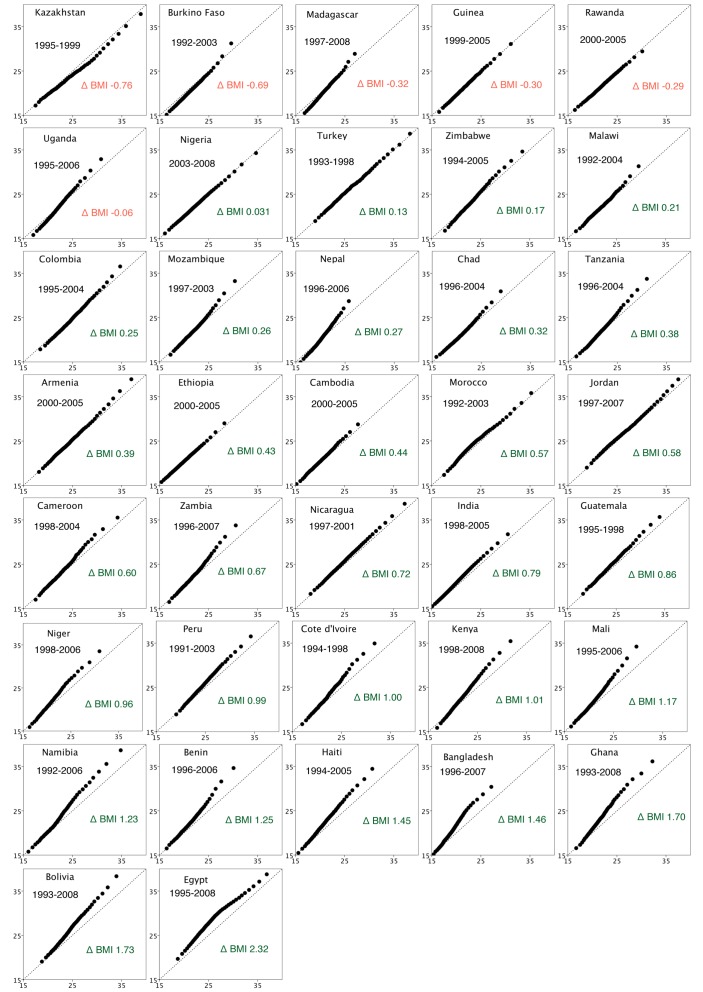
QQ plots of BMI in 37 countries. Country plots are ordered in terms of increasing mean weight between baseline and final survey, from smallest to largest. *x*-axis BMI at the baseline survey; *y*-axis BMI at final survey cycle. The diagonal line *y* = *x* is the line of equality between baseline survey and final survey. Δ BMI, change in mean BMI from first survey to last survey. Red text indicates decline in mean BMI; green text indicates increase in mean BMI.

Figure 2 shows the QQ plot of BMI for each country comparing the baseline survey to the most recent survey cycle. The charts are ordered by the degree of increase in mean BMI between these survey cycles, from Kazakhstan to Egypt. As countries show progressively larger increases in their mean BMI between cycles, a pattern of divergence emerges in the QQ plots. Lower percentiles of BMI remain closely distributed over the baseline survey; in contrast, there is a divergence of BMI at higher percentiles, representing increased weight gain in this segment of the BMI distribution. This change is visually represented in QQ plots by an increased distance between points at higher percentiles and the line *y* = *x*. The weight gain is not equally shared across the full distribution of BMI values in any country (this would be visually represented by a uniform linear shift off the line *y* = *x*).

**Figure 3:**
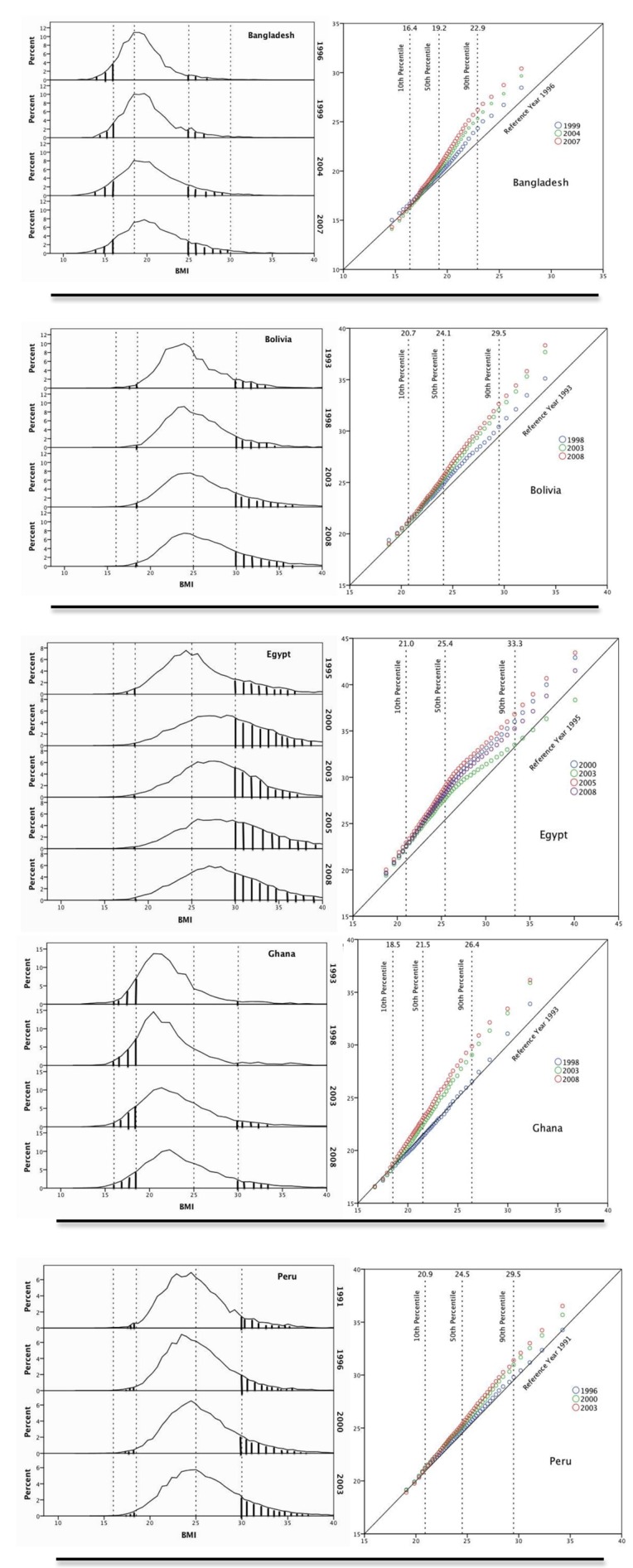
Population distribution of BMI over time in Bangladesh, Bolivia, Ghana, Peru, and Egypt. BMI histograms (panels on left). Vertical reference lines (dotted) represent established thresholds for BMI: 16.0 (chronic energy deficiency), 18.5 (underweight), 25.0 (overweight), and 30.0 (obese). Shaded regions represent proportion with BMI<16.0 or >25.0 in Bangladesh and proportion with BMI <18.5 or >30.0 in Bolivia, Ghana, Peru, and Egypt. QQ plots (panels on right). *x*-axis BMI at the baseline survey. *y*-axis BMI at successive survey cycles. The diagonal line *y* = *x* is the line of equality between baseline survey and subsequent survey. Vertical reference lines (dotted) represent the 10th, 50th, and 90th percentile, with the value of BMI at the baseline survey at the top of each line.

In order to explore the pattern of changes in the QQ plots in greater resolution, the five countries with the most repeated surveys were examined across all survey cycles (Figure 3). For each histogram a matching QQ plot is shown. In each country there is an extension of the rightward tail of the BMI histogram in successive surveys, with relative preservation of the proportion of the population at lower BMI. For Bangladesh, which has a low baseline mean BMI of 19.5 kg/m^2^, this can be visualized by examining the proportion of the population below a BMI of 16.0 kg/m^2^ versus the proportion greater than 25.0 kg/m^2^. In Bolivia, Ghana, and Peru, a similar pattern emerges when examining the proportion below 18.5 versus the proportion over 30.0. Egypt showed a slightly different pattern of change then other countries in the DHS, with greatest weight gain possibly occurring along the middle of the BMI distribution.

## Discussion

Our study of changes in the BMI distribution among women in LMICs has two salient findings. First, increases in mean BMI across populations, and within populations between cross-sectional surveys conducted at different time points, is associated with an increase in the degree of spread (or dispersion) of BMI values. Second, the pattern of weight gain differs markedly between segments of the population, with higher BMI segments gaining weight at an increased rate relative to lower BMI segments. As the average BMI in a population increases, the prevalence of overweight and obese is increasing at a much faster rate than the decline in the prevalence of underweight.

These results suggest that the concept of “average weight gain” of a population in LMICs belies an unequal distribution of how this change is occurring. As populations gain weight there is widening inequality in how that weight gain is distributed—for every 1.0 kg/m^2^ increase in mean BMI, the relative rate of increase in overweight is approximately 60% and increase in obese approximately 40% greater than the decline in underweight. Examining the tails of the BMI distribution, for every 1.0 kg/m^2^ increase in median BMI there is an increased spread between the 5th and 95th percentiles and resulting flattening of the BMI distribution, with the 95th percentile increasing at approximately 2.5 times the rate of 5th percentile. This finding has particular relevance given the possible J-shaped relationship between BMI and mortality, with increased mortality risk at both low BMI and high BMI levels, especially in low-income countries [25]–[28]. In this current study, underweight segments of the population that are at increased risk due to their low body weight, and who may benefit from weight gain, show a proportionally smaller increase in BMI over time relative to the population aggregate gain. In contrast, overweight and obese segments of the population at increased risk due to their already high baseline BMI, are expanding in prevalence over subsequent surveys. It is also important to note that the prevalence of low BMI remains very high in many low-income countries, with a maximum prevalence of underweight of 27% in Bangladesh in the most recent survey cycle of the DHS (Table 2). The patterning of BMI with wealth, has been previously documented in the LMICs studied in this analysis, and this may be contributing to the relatively slow increase in weight over survey cycles at the low end of the BMI distribution [12],[29]. Relatively larger weight gain in higher segments of the BMI distribution may represent a self re-enforcing cycle of physiologic, socioeconomic, and psychosocial factors [30].

Prior analyses examining BMI trends in the DHS primarily studied within-country effects of socioeconomic factors, cross-national effects of macroeconomic factors, and secular changes in prevalence of BMI categories [2],[11],[12],[29],[31],[32]. The major finding from these papers was that most countries had a rapid rise in overweight prevalence, more moderate decline in underweight prevalence, and strong socioeconomic patterning in how these changes occurred. This current analysis differs significantly in focus from prior work, studying how BMI has changed across the entire distribution between surveys and showing that summary measures provide a relatively limited picture of change at both the low weight and high weight segments of the population. Studies examining mean changes in BMI or percent underweight/overweight by socioeconomic status also capture the differential changes in the population distribution of BMI [10]–[12],[18],[29], albeit by *a priori* defining the groups indicating the association of measures of socioeconomic status with degree of weight gain.

The widely varying pattern of weight gain demonstrated in this paper calls into question the ability of limited measures of a population (such as mean BMI, or prevalence of overweight/obese) to accurately portray true population-level change. We show here that the change in average BMI is not equal to the degree of change at both ends of the BMI distribution, and therefore a single parameter in isolation is misleading. A more accurate analysis of how populations change over time requires information about both the center of the population and its extremes. This finding has implications for analyses that summarize global population change via mean BMI or overweight/obese, and may have implications for many other risk factors such as blood pressure, cholesterol, or fasting glucose that are similarly analyzed [1],[33]–[35]. We suggest that a more comprehensive picture of true global patterns of weight gain must examine those at the lower end of the BMI distribution, not just those at the center or upper extremes.

**Figure 4:**
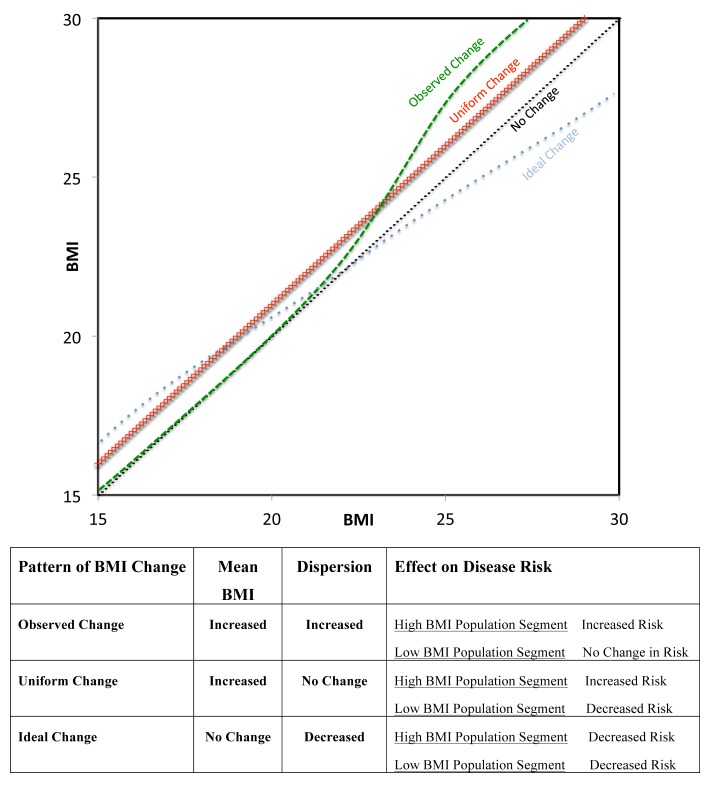
QQ plots of different models of population change in BMI over time. The observed pattern of change (from Figure 2) in green, uniform change model in red, and a proposed model of ideal change in blue. The corresponding table indicates impact of each model of change on (1) mean BMI, (2) dispersion of BMI, and (3) risk level of low and high BMI population segments.

Rose demonstrated that if the spread of a risk factor (e.g., blood pressure) distribution remains constant as the mean increases, the mean level of that risk factor predicts the prevalence of people who are classified as sick (e.g., hypertensive). He further hypothesized that population change could be summarized by following the change of the mean [3]. We show here that BMI in women in LMICs does not follow this pattern of change both across countries, and within countries between surveys conducted at different times. This finding has implication for the population approach to prevention since promoting a change in mean BMI appears to affect people who are underweight and overweight in very different ways. For example, we show that an increase in mean BMI can result in relatively small decreases in underweight relative to larger increases in overweight and obese. In Figure 4, we have described different patterns of BMI change in a population. The actual pattern of weight gain demonstrated here is plotted in green, with very little weight change in low BMI percentiles and increased weight gain in high BMI percentiles. This pattern would result in an increase in mean BMI and in dispersion, and would be detrimental to both high weight (due to increased risk) and low weight (due to persistent risk) segments of the population. Rose's model of uniform population change is plotted in red, with increasing mean BMI and preserved SD. This scenario may lead to improved health in underweight segments but worsening health in high weight individuals. Finally, we propose a model of population change that would have ideal characteristics (blue). There would be an increase in weight among the underweight and decrease in weight among the higher segment of the BMI distribution. This model could lead to both a reduction in SD and no change in mean BMI, and provides a direct example of a situation where examining mean BMI alone would be misleading about the change in population level risk. This model of ideal change would result in health gains for both extremes of the distribution. Notably, a population-based approach that simply relied on changing the mean BMI in a population would not be able to achieve this dual benefit. The specific public health intervention by which this ideal change could be achieved is unclear given the patterns observed in this paper, and it is possible that rather than a single broad population strategy, what would be required are targeted interventions to reduce weight in high BMI segments of the population and to increase weight in low BMI segments.

This study has a number of limitations. First, it is based on repeated population surveys and therefore cannot establish weight gain within an individual over time. However, our goal is to examine patterns of change at the population level and therefore the ability to detect change within individuals is less relevant. To our knowledge no nationally representative cohort studies with repeated measurements exist across such a broad range of countries and this should be a focus of future research. The DHS may represent the best currently existing source of data for examining population level changes globally. Second, only women aged 20–49 in LMIC were examined in the DHS. Data in the United States suggest similar patterns of change in men and women in the distribution of BMI, but do not show markedly unequal patterns of weight gain across the BMI distribution [9]. Whether unequal weight gain occurs across segments of the BMI distribution should be examined in both men and women and across a broader range of countries in future studies. Third, BMI may not be the best measure of adiposity with respect to the risk of important outcomes such as cardiovascular disease [36], and the DHS are limited by the absence of measures of abdominal obesity such as waist-to-hip ratio. Fourth, the limited number of survey cycles within most countries in the DHS only allowed fitting with a slope fixed effect multilevel model. Future studies, with a greater number of data points within countries, should examine whether a random effects model provides further insight into varying patterns of weight gain globally. Fifth, survey samples were not always the same size within the countries and the time period between surveys was not consistent across countries. Finally, the DHS does not allow us to test the mechanism driving the divergence patterns we observed and this is an important area for future research.

In summary, we show that increases in mean BMI have been associated with increased spread in levels of BMI across and within populations. Reliance on mean BMI or overweight/obese to represent population level change does not capture the rapidly increasing BMI among high percentile segments of the BMI distribution and relative stagnation of body weight among low percentiles of the distribution. Studies that characterize populations, and their change over time, should not rely on limited measures of the centrality or deviance but should examine patterns of change across the entire distribution.

## Abbreviations

BMI: body mass index
DHS: Demographic Health Surveys
LMIC: low- and middle-income country
QQ: quantile-quantile
SD: standard deviation
